# Controlling Properties and Cytotoxicity of Chitosan Nanocapsules by Chemical Grafting

**DOI:** 10.3390/md14100175

**Published:** 2016-09-30

**Authors:** Laura De Matteis, Maria Alleva, Inés Serrano-Sevilla, Sonia García-Embid, Grazyna Stepien, María Moros, Jesús M. de la Fuente

**Affiliations:** 1 Instituto de Nanociencia de Aragón (INA), Universidad de Zaragoza, Edificio I+D, calle Mariano Esquillor s/n, 50018 Zaragoza, Spain; mariaalleva1234@gmail.com (M.A.); inessersev@gmail.com (I.S.-S.); sonia.garcia.embid@gmail.com (S.G.-E.); gstepien@unizar.es (G.S.); 2 Instituto de Ciencia de Materiales de Aragón (ICMA), CSIC-Universidad de Zaragoza, Edificio I+D, calle Mariano Esquillor s/n, 50018 Zaragoza, Spain; 3 Istituto di Scienze Applicate e Sistemi Intelligenti “E. Caianiello”, Consiglio Nazionale delle Ricerche, Pozzuoli 80078, Italy; m.moros@isasi.cnr.it

**Keywords:** chitosan, hydrogel, surface grafting, nanocapsules, stability

## Abstract

The tunability of the properties of chitosan-based carriers opens new ways for the application of drugs with low water-stability or high adverse effects. In this work, the combination of a nanoemulsion with a chitosan hydrogel coating and the following poly (ethylene glycol) (PEG) grafting is proven to be a promising strategy to obtain a flexible and versatile nanocarrier with an improved stability. Thanks to chitosan amino groups, a new easy and reproducible method to obtain nanocapsule grafting with PEG has been developed in this work, allowing a very good control and tunability of the properties of nanocapsule surface. Two different PEG densities of coverage are studied and the nanocapsule systems obtained are characterized at all steps of the optimization in terms of diameter, Z potential and surface charge (amino group analysis). Results obtained are compatible with a conformation of PEG molecules laying adsorbed on nanoparticle surface after covalent linking through their amino terminal moiety. An improvement in nanocapsule stability in physiological medium is observed with the highest PEG coverage density obtained. Cytotoxicity tests also demonstrate that grafting with PEG is an effective strategy to modulate the cytotoxicity of developed nanocapsules. Such results indicate the suitability of chitosan as protective coating for future studies oriented toward drug delivery.

## 1. Introduction

In the last few decades, many kinds of nanocarriers have been developed for delivery and targeting of therapeutic or diagnostic agents, thanks to some important advantages that they offer depending on their physico-chemical properties [1,2].

Depending on the specific needs, the nanocarrier type and formulation process must be chosen on the basis of therapy goals and administration route [3]. The most common nanocarriers can be classified as follows: solid (inorganic or organic) nanoparticles [4,5], nanospheres (polymeric matrices or hydrogels) [6,7,8], or nanocapsules (usually liposomes, emulsion-based, or protein-based nanocapsules) [9,10,11].

According to Vrignaud and co-workers, nanocapsules are vesicular systems, composed of an oily or an aqueous core that can be considered as a reservoir in which the drug is confined to a cavity, surrounded by a polymeric shell [12]. Nanocapsules can be obtained combining nanoemulsion and a polymeric coating. Nanoemulsion particles are stable colloidal suspensions obtained by mixing an organic phase containing oil and a lipophilic surfactant with an aqueous one containing a hydrophilic surfactant, resulting in a particle size ranging from 20 to 600 nm. The characteristics of the obtained particles depend on the spontaneity of the emulsification process that is affected by the nature of the single components of the reaction mixture and also by the rate of the mixing process [13,14]. Depending on the desired application, a coating is necessary to further stabilize the nanoscaled particles resulting from this spontaneous process and improve surface properties. The most commonly used coatings are natural polymers, which are deposited on the nanoemulsion template surface to produce a rigid and dense shell [15]. The shell can be easily tailor-made to achieve desired characteristics and its surface chemistry can be tuned to obtain a proper functionalization for biological targeting [16,17,18]. 

Natural polymers are among the most used for these kind of coatings since they usually provide a high colloidal stability in water suspensions. Active research is now focused on the use of hydrophilic biopolymers as carrier coatings because of their biocompatibility and biodegradability [19,20]. Chitosan (CS) has been used for the development of sustained release carriers, mucoadhesive formulations, and peptide drug absorption systems [21,22,23,24]. It is currently employed to prepare nanomaterials with mucoadhesive properties since its positive charges allow the interaction of particles with the negative charge of mucin, resulting in a better interaction with mucosal tissues and with epithelial cells. Moreover, it is known that the positive charge of the polymer can promote the paracellular transport by tight-junction regulation [25,26].

In this work, core-shell nanocapsules made of a nanoemulsion core and a chitosan shell were synthesized and characterized with the aim of obtaining a multipocket nano-reservoir carrier to be used in future applications for sustained release of different drugs. The secondary effects—toxicity, poor solubility, and bioavailability—of new drugs lead to the need of their encapsulation to protect them from degradation and to enhance their stability and solubility [27,28].

Thanks to the presence of chitosan amino groups, the surface of the obtained nanocarrier can also be grafted with specific moieties in order to tune the net charge to introduce specific functional groups and/or improve the carrier stability in biological media and physiologic solutions for intravenous administration [29,30,31,32,33]. The development of a smart nanocarrier is strictly related with controlling its surface properties since they are responsible for specific recognition of targeted sites but also for non-specific adsorption of serum proteins. A decrease in protein adsorption leads to a reduced uptake by the mononuclear phagocytic system, leading to a prolonged circulation time in the blood stream and to a higher residence time of the encapsulated drug. Poly (ethylene glycol) (PEG) coatings are known to prevent aggregation and serum protein adsorption by steric and hydration repulsions leading to more stable colloidal suspensions of nanocapsules in physiological media [33,34,35]. In this work, the surface of the developed chitosan-coated nanocapsules was grafted covalently with PEG molecules through a novel, simple, and reproducible strategy based on the use of a homobifunctional crosslinker, bis (sulfosuccinimidyl) suberate (BS^3^), that links aminated PEG molecules to amino groups on nanocapsule surface. Nanocapsule behavior in different media was evaluated in terms of aggregation degree before and after grafting and the effect of PEG on their cytotoxicity was also assessed.

## 2. Results

### 2.1. Chitosan-Coated Nanocapsules

A nanoemulsion method was developed to obtain small nanoparticles (smaller than 200 nm) to be used as a template for the following polymer coating and reinforcement. The aim was to obtain capsules with a lipophilic core and a hydrophilic cationic shell of chitosan hydrogel.

The synthesis of the nanocapsules was carried out in two steps. The formation of the nanoemulsion template particles was carried out simply by adding a water-miscible organic solution of Span^®^ 85/oleic acid (Croda International PLC, Cowick Hall Snaith, Goole, East Yorkshire, UK) to a Tween^®^ 20 (Croda International PLC, Cowick Hall Snaith, Goole, East Yorkshire, UK) aqueous solution under stirring. Optimal ratios between components have been found adapting a method reported by Bouchemal et al. [14]. The particles are immediately and spontaneously formed. Chitosan has been chosen for the coating of the nanoemulsion template since it is one of the richest in amino groups’ natural polymers.

The presence of such functional groups is responsible for the ability of the polymer to gelify in presence of multi-anions leading to the formation of hydrogel. Moreover, the amino groups exposed on hydrogel shell surface allow the easy functionalization of the nanocapsule with the desired moiety.

Chitosan is directly added to the nanoemulsion that is subsequently mixed with a sodium sulfate solution to obtain the coating with a chitosan shell. This treatment has been used in several works to obtain chitosan particles [36,37,38]. In our case, the method allowed to obtain a hydrogel polymer shell as a result of the interaction of chitosan polyelectrolyte structure with sodium sulfate. Sodium sulfate acts as a bridge promoting interactions between polymeric chains.

The result of the synthesis is a water-stable suspension of chitosan-coated nanocapsules (CS-NCs).

**Figure 1:**
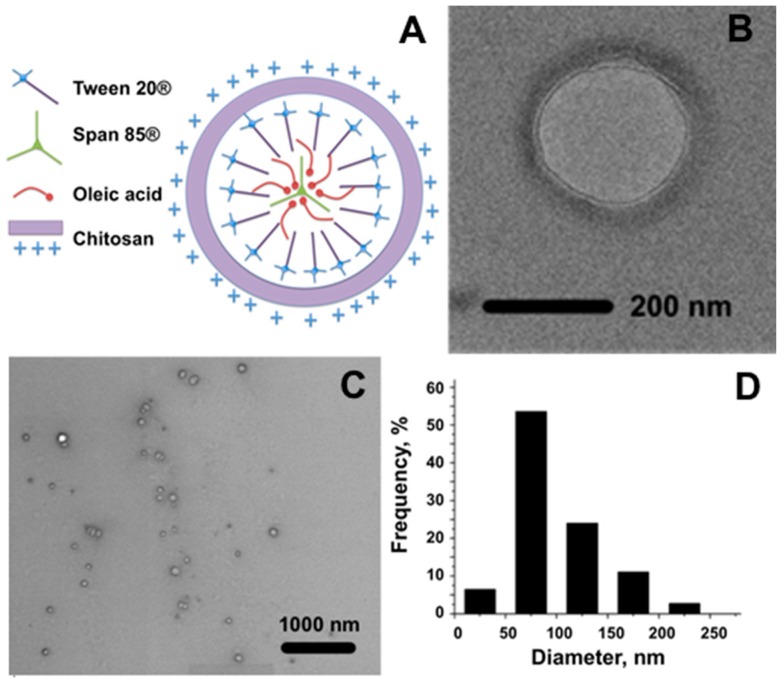
Morphological characterization of chitosan-coated nanocapsules (CS-NCs). (**A**) Schematic representation of a section of a nanoemulsion-based and chitosan-coated nanocapsule; (**B**) Bright Field Transmission Electron Microscopy (BF-TEM) image and (**C**) Environmental Scanning Electron Microscopy (ESEM) image of nanocapsules from a section of the epoxy resin block; (**D**) representation of frequency count analysis of size distribution.

A schematic representation of the hypothesized nanocapsule structure is reported in Figure 1A. Moreover, to investigate the morphology of the obtained material, an electron microscopy characterization was carried out on chitosan-coated nanocapsules using both Bright Field Transmission Electron Microscopy (BF-TEM) and Environmental Scanning Electron Microscopy (ESEM) (Figure 1B,C respectively). Due to the sensitive nature of the sample common in soft materials, previous fixation, dehydration, dyeing and resin embedding were necessary.

The sample turned out to be composed of spherical capsules of a quite homogeneous size. The polymeric shell can be appreciated in BF-TEM and ESEM images (Figure 1B,C). ESEM estimation of the diameter distribution is reported in Figure 1D. The number of capsules of different sizes, as percentages over the total number of measured capsules, is reported in the graph as a function of the diameter. The calculated mean diameter of NCs is 104 nm.

**Figure 2:**
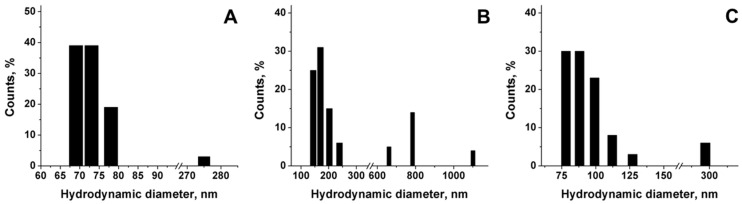
Dynamic Light Scattering (DLS) measures reported as percentages of frequency counts at each hydrodynamic diameter: nanoemulsion before chitosan coating (**A**); nanocapsules obtained by sonication (sCS-NCs) (**B**); and non-sonicated nanocapsules obtained by stirring (nsCS-NC) (**C**).

As optimization of the process, the importance of the sonication treatment during hydrogel shell formation was evaluated by substituting it with a gentle stirring while adding nanocapsules to Na_2_SO_4_ solution. The elimination of the sonication step in the synthesis process could represent an advantage in terms of applicability of the process for future applications to industrial production with high levels of scale-up. Nanocapsules obtained with the modified process have been characterized in terms of hydrodynamic diameter and surface charge and they have been compared with the sonicated ones. In Figure 2, Dynamic Light Scattering (DLS) measures of the hydrodynamic diameter of the nanoemulsion template just before chitosan coating (A), nanocapsules obtained by sonication (sCS-NCs) (B), and non-sonicated nanocapsules obtained by stirring (nsCS-NC) (C) are reported and compared.

Hydrodynamic diameter of chitosan–coated nanocapsules always increases with respect to the nanoemulsion template (Figure 2A). Nevertheless, in the case of sonicated capsules the final hydrodynamic diameter is much higher than the non-sonicated sample (Figure 2B). The value is also higher than the diameter distribution obtained from SEM images referring to sonicated nanocapsules. Moreover, the presence of a significant percentage of big aggregates is observed in the sCS-NC. The increase in the diameter observed can be attributed to aggregation phenomena, indicating a lower stability of this sample in water suspension. In the case of non-sonicated samples, a certain amount of aggregates are detected too, even though such aggregates have a smaller diameter than the ones obtained by sonication. In any case, the presence of a low percentage (i.e., 5%–10%) of aggregates could be considered acceptable for future purposes. The hydrodynamic diameter of the nanocapsules stored in water suspension has been found to be reproducible over at least two months, indicating the suitability of chitosan to successfully stabilize the nanoemulsion.

It is supposed that the hydrogel formation treatment affects the properties of the polymer-coated surface of the nanocapsules depending on the degree of the interaction that could be established between –NH_2_ and –OH on polymer chains and Na_2_SO_4_ molecules. Moreover, it should be taken into account that the presence of salts can also promote hydrophobic interactions between polymer chains themselves. As a consequence of the establishment of such hydrophobic interactions, the exposure amino groups on the surface of the nanocapsule would be favored. To evaluate how the sonication during hydrogel formation can improve the interactions and so affect the nanocapsule surface properties, sonicated and non-sonicated nanocapsules have been compared in terms of surface potential. In particular, analysis of Z-potential and amino group spectrophotometric determination analysis are reported. Both capsules showed a positive potential when measured in a 10 mM KCl solution, being slightly more positive the surface of sCS-NC (+21.1 mV) than the surface of nsCS-NC (+13.8 mV). The observed variation in the surface potential depending on the sonication treatment could be explained in this case by the presence of a higher number of amino groups exposed on the outer surface of chitosan shell in the case of sCS-NC. This hypothesis was confirmed by the quantification of amino groups of the chitosan shell of nanocapsules by the spectrophotometric method of Orange II dye, previously reported and already optimized for inorganic nanoparticles [39]. Briefly, the method is based on a pH-dependent interaction between positively charged amino groups and –SO^3−^ group of Orange II dye. This spectrophotometric method is simple, inexpensive, and easy, and its most important advantage over other methods for amino quantification consists in the use of a molecule with low steric hindrance—an especially important aspect for porous materials materials. Moreover, the relationship between amino groups and reactant is in this case of 1:1, allowing a direct and reliable quantification [40]. In the present work, the method has been slightly modified to use syringe filters as support for the separation of nanocapsules from the solution during all the washing steps. Results from the spectrophotometric assay for the measurement of amino groups through the interaction with Orange II dye also proved the presence of a high number of positively charged amino groups in the case of sCS-NC (0.35 μmol·mg^−1^) while a value of amino groups of only 0.2 μmol·mg^−1^ was obtained in the case of nsCS-NC.

It should be taken into account that results from Orange II interaction only represented the amount of amino groups available for the interaction with dye molecules and that the amount of these groups could be lower than the total amount of –NH_2_ of the nanocapsules. Data from the Orange II assay were in good agreement with Z potential analysis. sCS-NC presented a number of moles mg^−1^ of –NH_2_ groups almost double with respect to the moles mg^−1^ of –NH_2_ of nsCS-NC.

Thermo Gravimetric Analysis (TGA) of chitosan-based nanocapsules sonicated or not during the synthesis process is reported in Figure S3 of the Supplementary Materials. The weight loss corresponding to chitosan shell can be appreciated at ~200 °C and it corresponded to 50% weight of the sCS-NC. Surprisingly in the case of nsCS-NC this percentage is even higher, where chitosan represents 65% of the total weight of the sample. This evidence demonstrated that the observed decrease of amino groups on the surface of non-sonicated nanocapsules was not due to a decrease in the chitosan total amount. Consequently, it is reasonable to suppose that the organization of chitosan layer and interactions between polymer chains themselves and with the surface of the nanoemulsion template are slightly different depending on the condition applied during the synthesis process.

### 2.2. Grafting of the Surface of Chitosan-Coated Nanocapsule

PEG coating has been chosen in this work for the further optimization of the carrier. As previously stated in the introduction and well documented in the literature, the prevention of unspecific adsorption of serum proteins on carrier surface represents a very important goal to allow a prolonged circulation time in the blood stream. From this point of view, pegylation is reported to be one of the most successful strategies [33,34,35].

**Scheme 1:**
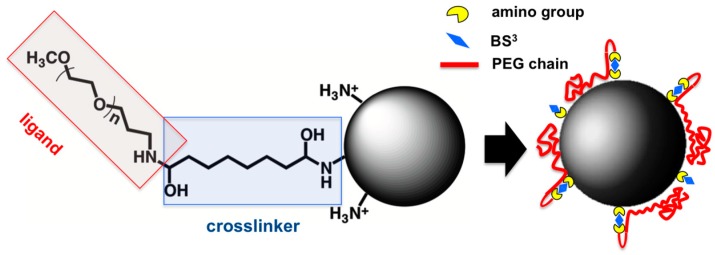
Chitosan-coated nanocapsule grafted with aminated polyethylene glycol (PEG) through BS^3^ linking.

Nanocapsules obtained without sonication during the synthesis have been selected for the process of grafting of the surface due to their better stability in water suspension. The surface was grafted with α-methoxy-ω-amino poly (ethylene glycol) (5000 Da) (aminated PEG) through a previous reaction with the homobifunctional linker bis (sulfosuccinimidyl) suberate (BS^3^) (Scheme 1). As the colloidal stability of nanocapsules strongly depends on their surface properties, which in turn greatly affect nanocapsule cytotoxicity in animal cells, the possibility of obtaining nanocapsule surface with a low-density coverage of PEG molecules (5 nmol/mg initially added) (ldCS-NC) and a high-density coverage (100 nmol/mg initially added) (hdCS-NC) was explored. Consecutively, the final properties of the obtained materials were studied. A schematical representation of the strategy used for grafting is reported in Scheme 1.

The determination of the accessible amino groups was fundamental for the optimization of the grafting protocol. In fact, the method utilized here implied the use of a homobifunctional linker in which the main drawback lays in the possibility of crosslinking of amino groups from different nanocapsules and production of aggregates if the amount of reactants is not strictly controlled. For this reason, the value of accessible amino groups has been used as the starting point to adjust the amount of reactants in the following steps of the process. In particular, the amount of BS^3^ has been always maintained below the total moles of functional groups of nanocapsules to reduce the possibility of crosslinking between capsules. To complete the process, the amount of aminated PEG exceeded twice the used amount of BS^3^ in all the experiments to assure the reaction with all the reactive groups of BS^3^ on the surface.

The presence of PEG on nanocapsule surface has been evaluated by Fourier Transform Infrared Spectroscopy (FTIR) analysis (data reported in Supplementary Materials). The properties of chitosan-coated nanocapsules before and after the grafting have been compared and the FTIR spectra of all the single components of nanoemulsion-based nanocapsules are reported respectively in Figures S4 and S5 of the Supplementary Materials.

In the FTIR spectrum of nsCS-NC (Figure S4A), it is possible to recognize the presence of the four starting components, meaning that the final composition is compatible with the hypothesized structure of the nanocapsule (Figure 1A). Moreover, reported in Figure S6 of the Supplementary Materials a comparison of sCS-NCs and nsCS-NC demonstrates that the sonication process is not affecting the chemical interaction between nanoemulsion template and chitosan shell.

The grafting process consists of three steps. The first one is the incubation with the linker to provide the surface with the sulfosuccinimidyl ester group sensitive to the linking of PEG. During the second step the ligand is added and finally the grafted nanocapsules are incubated with Tris-HCl buffer to quench the free sulfosuccinimidyl ester groups eventually not linked to any PEG molecule. This last step is fundamental to avoid the crosslinking between nanocapsules due to the reaction between free sulfosuccinimidyl ester groups and amino group on a different capsule.

To determine if the grafting with PEG successfully occurred, the FTIR spectrum of the intermediate step of nanocapsules after the incubation with BS^3^ is also reported (Figure S4B). It should be noted that at this intermediate step the sample is incubated with Tris-HCl buffer after the linking of BS^3^ to avoid the crosslinking with amino groups on other nanocapsule surface through the reactive groups still available for ligand reaction. In the case of samples incubated with ligand in the second step, Tris-HCl was added at the end of the process (after incubation with the ligand) but in this case it can be supposed that BS^3^, which should have reacted previously with PEG, is not available to react with Tris. This hypothesis was confirmed by comparing FTIR spectra of nanocapsules after the incubation with BS^3^ (Figure S4B) and spectra in Figure S4C,D referring to nanocapsules grafted with a low density and high density of PEG, respectively. Peaks at 3180 and 3100 cm^−1^, as well as peaks at 1630 and 1550 cm^−1^ referring to Tris are present in the intermediate step and (in ldCS-NC with lower intensities) but they completely disappeared in hdCS-NC spectrum.

Differences in the spectra could also be appreciated, comparing peaks and intensities ratios between 1150 and 1030 cm^−1^. In this region the C-O-C peak (1105 cm^−1^) presents an increased intensity, compared to other peaks in the same region, especially in the hdCS-NC spectrum. Moreover, in this sample peak at 1055 cm^−1^ is lost. This peak is supposed to refer to R-SO^3−^ group that is released from BS^3^ in the linking reaction. In the BS^3^ intermediate step the peak is still appreciable and it can be observed as a low intensity peak in ldCS-NCs too. It is possible that in these samples not all BS^3^ reactive groups are quenched after Tris-HCl incubation and that some of them are still present on the surface. In any case this state did not represent a problem for the quality of the sample since any appreciable crosslinking and consequent aggregation are observed.

The successful grafting on nanocapsule surface was proved also by measuring the Z potential of the material surface before and after the modification with PEG. Using both a low amount and high amount of PEG, a decrease in the potential was observed, indicating a decrease in the free amino groups on the surface and an increase in the total electronegativity of the surface.

A confirmation of this evidence is the small difference in the decrease of amino groups (measured by Orange II spectrophotometric assay) registered between low-density and high-density PEG-covered nanocapsules. The measured amounts of amino groups are 0.1 and 0.08 μmol/mg respectively, corresponding to 50% and 40% of the amounts of amino groups on ungrafted nanocapsules. These values are considered as the apparent disappearing of amino groups probably due to a screening effect for the presence of PEG molecules on the surface. Orange molecule interaction with –NH_3_^+^ groups would be hampered by the presence of the polymer chains since, in the case of low-density PEG coverage, it can be supposed that polymer molecules stand in a conformation that lays around nanocapsule surface, probably thanks to a possible hydrogen bond interaction of the PEG chain with positively charged amino groups on the nanocapsule surface (see Scheme 1). The tendency to form such interactions could also be responsible for a lower than expected efficiency of linking in the case of high-density coverage sample.

**Figure 3:**
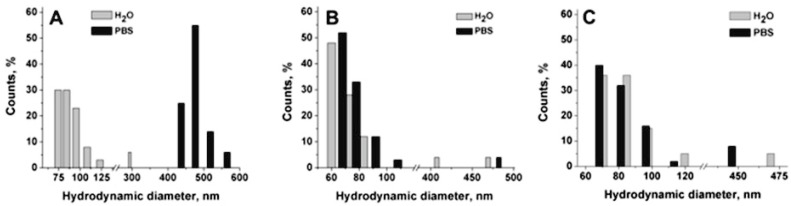
Hydrodynamic diameters of chitosan-coated nanocapsules before (**A**) and after grafting with different amounts of PEG ((**B**) and (**C**), respectively referring to low-density coverage (ldCS-NC) and high-density coverage (hdCS-NC)).

The effect of the grafting on the behavior of nanocapsules in physiological medium was further evaluated by measuring the degree of aggregation of grafted and not-grafted nanocapsules in water and phosphate saline buffer (PBS) by means of hydrodynamic diameter measurement. Ungrafted nanocapsules are sensitive to the presence of salts in the medium and they tend to aggregate during incubation in physiological media like PBS. The grafting with PEG chains is a common strategy to improve the stability of nanoparticles in physiological and in vitro culture media [33]. Both phosphate ions and proteins can adsorb onto nanocapsule surface due to the presence of amino groups, producing the crosslinking between different capsules. Moreover, the presence of salts can produce aggregation due to a salting-out-like effect. The presence of PEG on nanocapsule surface would screen the amino groups on the surface from interaction with salts in the medium. All samples were measured in water and PBS and their diameters were compared to assess the effect of PEG on prevention of aggregation (Figure 3).

In Figure 3A, a strong aggregation effect in PBS is reported for ungrafted chitosan-based nanocapsules. The mean hydrodynamic diameter changed from 103 nm in water to 471 nm in PBS. On the contrary, the diameter and the PDI of grafted nanocapsules are maintained in PBS, even in the case of ldCS-NCs, confirming that the grafting successfully occurred in both cases and that it was effective for nanocapsule stabilization.

To further demonstrate the stabilizing effect of PEG grafting on nanocapsule surface, an aggregation test has been carried out by measuring the hydrodynamic diameter of grafted and ungrafted nanocapsules in the presence of increasing concentrations of Bovine Serum Albumin (BSA). As in the case of the above reported stability assay in presence of PBS, the high density of amino groups on nanocapsule surface can be considered responsible for the adsorption of proteins and aggregation observed. 

**Figure 4:**
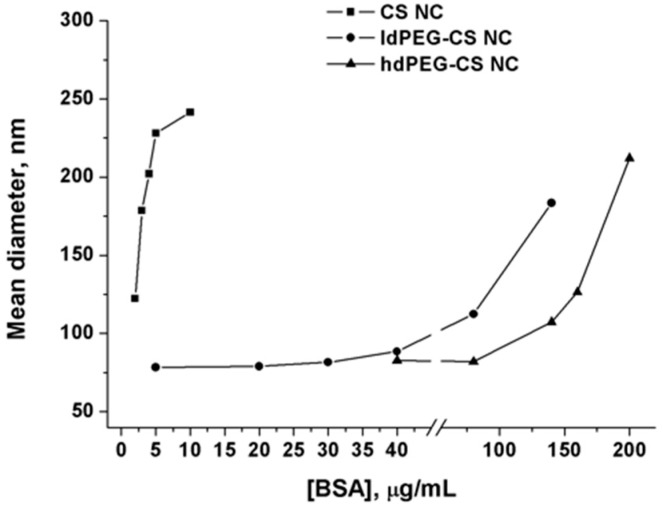
Hydrodynamic diameters of chitosan-coated nanocapsules before and after grafting with different amounts of PEG measured in presence of different concentrations of Bovine Serum Albumin (BSA).

The comparison of the behaviors of ungrafted nanocapsules, ldPEG-CS NC and hdPEG-CS NC, has been reported in Figure 4.

Data reported in the graph demonstrated without any doubt the strong stabilizing effect of PEG on nanocapsules in the presence of proteins. The grafting with PEG, and so the strong decrease of the free amino groups on the surface lead to a very good stability of the nanocapsules in a protein-rich medium. Both ldPEG-CS NC and hdPEG-CS NC showed a significant increase in the hydrodynamic diameter only at very high concentrations of proteins (higher than 0.1 mg/mL). Comparatively, the concentration of BSA one order of magnitude lower is enough to produce the same increase in the diameter in the case of ungrafted NCs.

### 2.3. Cell Viabily and Internalization Assays

**Figure 5:**
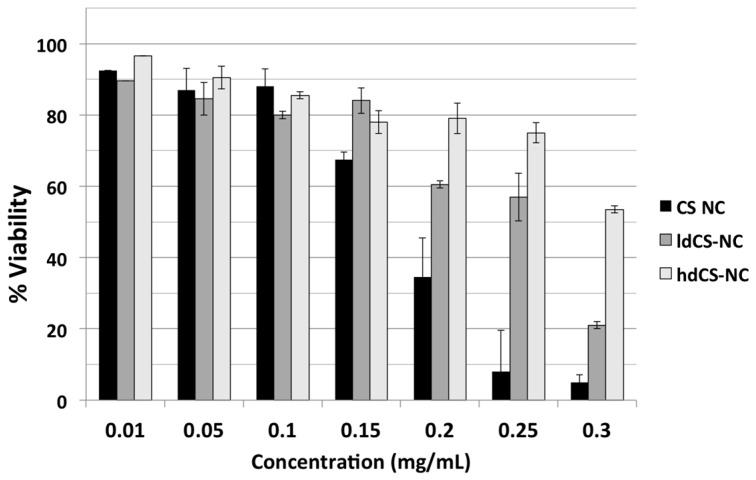
Viability test with chitosan-coated nanocapsules before and after grafting with low and high-density coverage of PEG.

The cytotoxicity of chitosan-based nanocapsules before and after the grafting was tested on Vero cells through MTT spectrophotometric assay. Cells were incubated for 24 h with different concentrations of nsCS-NC, selected as ungrafted nanocapsules over the sonicated ones due to their better stability in water, and with the same concentrations of ldCS-NC and hdCS-NC. In Figure 5, the comparison between the grafted nanocapsules and the ungrafted ones is reported in terms of percentage of viability of cell culture after 24 h incubation.

It can be observed from Figure 5 that the grafting significantly improved the safety of the nanocapsules, especially when using high concentrations of nanocapsules (greater than 0.15 mg/mL). Both types of grafted nanocapsules were less toxic to the cells, especially the high density ones (at high concentrations).

**Figure 6:**
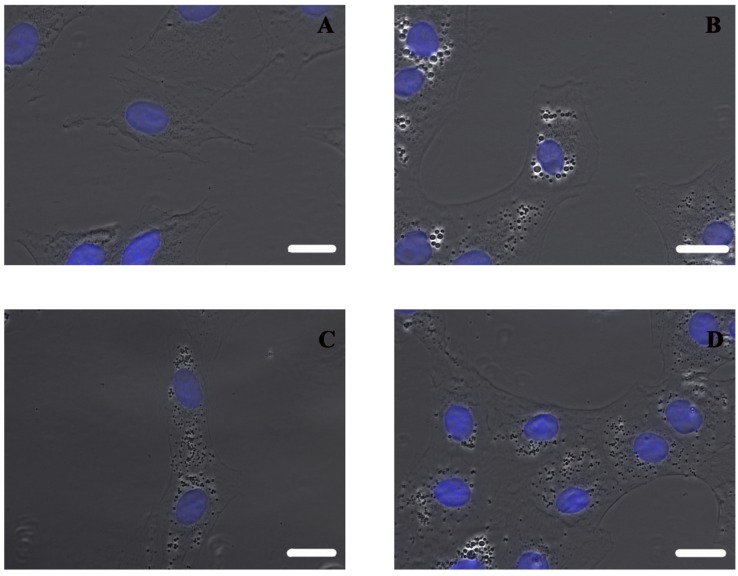
Inverted microscope images from (**A**) control cells; and cells incubated for 24 h with (**B**) non-sonicated nanocapsules; (**C**) ldCS-NC; and (**D**) hdCS-NC. Scale bars correspond to 20 μm.

Using an inverted microscope, it can be observed that non-sonicated nanocapsules lead to bigger vesicles inside cells than both types of grafted nanocapsules (Figure 6), which could be related with the higher toxicity).

The test proved not only that aminated PEG was definitively linked to nanocapsule surface, but also that the conformation of polymer chains obtained using the developed method of grafting for ldCS-NC hdCS-NC is effective for significantly improving the cytotoxicity of chitosan-based nanocapsules. Moreover, it should be noted that in the case of PEG-grafted nanocapsules the decrease in the cytotoxicity is still associated with a very high degree of internalization, indicating that the developed material can be considered a very effective carrier for drug delivery applications.

## 3. Discussion

Among all kinds of nanocarriers, nanocapsules are frequently the material of choice for biomedical applications since they offer the advantage of providing a good stability of encapsulated drugs and a favorable pharmacokinetic. The aim of the work was to develop nanocapsules to be used in the future as potential drug reservoir and whose composition could allow maximum flexibility of application together with great feasibility for the administration by different routes (intravenous injection, oral administration, and inhalation).

In this work, chitosan—a biocompatible polymer—has been used to obtain a potentially smart nanocarrier whose surface properties could be properly tuned depending on the desired application. A nanoemulsion-based nanocapsule has been used as template to be coated with a chitosan shell.

The process of chitosan hydrogel coating of a nanoemulsion template was studied in terms of exposure of amino groups on the surface depending on the interaction produced during the sonication process. It could be hypothesized that the sonication promoted interactions and produced stronger hydrophobic interaction between polymer chains, leading to a condensation of the structure that would lead to a higher exposure of amino groups and so to a higher availability for the interaction with the dye. On the other hand, it was also supposed that a different amount of polymer could be incorporated on the surface of the nanoemulsion template when sonication is used during the synthesis, leading to a different total amount of glucosamine units (and so amines) in the shell. The characterization of the material and the comparison between the sonicated and not sonicated one alloowed finally to confirm that the sonication process lead to a higher amount of amino groups exposed on nanocapsule surface.

Chitosan shell offers the possibility of an easy functionalization through amino groups on its surface so chitosan-based nanocapsules have been further coated with PEG since this is known to be a very effective strategy to stabilize surfaces in biologically relevant media. The possibility to tune the surface properties by varying the PEG density coverage has been explored and results obtained are compatible with a conformation of PEG molecules laying adsorbed on nanoparticle surface after covalent linking through their aminated terminal.

It is reported in literature that the attempt to produce high-density coverage of surfaces with PEG can result in a loss of efficiency of grafting. In fact, similarly to what reported for the grafting of other surfaces, it is reasonable to suppose that not all PEG molecules react immediately with BS^3^ linker on nanocapsule surface. Once they are linked, their tendency is to assume a mushroom-like conformation until the density of PEG molecules on the surface does not reach a value high enough to produce a change into a brush-like conformation [41]. In the case of high-density PEG-covered nanocapsules, the decrease in amino groups on the surface was found to be not proportional to the reactants, differently to what happened in the case of low-density ones, indicating that eventually a conformation of PEG molecules laying adsorbed on nanoparticle surface was hampering the grafting of masked amino groups.

Despite the fact that the obtained grafting degree was slightly lower than the one expected, a highly improved stability in physiological medium (resistance to salting out effect) was observed with both low and high PEG-covered nanocapsules (although at different degrees) indicating that the presence of PEG on the surface will allow the use of the grafted capsules in physiological media for biomedical applications.

We also demonstrated that the presence of PEG on chitosan surface of nanocapsules was effective in avoiding the unspecific adsorption of proteins. This issue is of the utmost importance as, following intravenous injection, non-passivated nanoparticles tend to adsorb plasma proteins (protein corona), changing the charge and size of them. This binding of plasma components will be responsible of the final fate of the nanocapsules, and can greatly influence the biodistribution and therapeutic efficacy [42]. To date, PEG is the most widely used polymer to prevent this protein corona formation, although the final effect depends on different parameters such as the molecular weight or grafting density [43].

Following the encouraging results obtained in physiological media and protein-rich media, preliminary tests with cell cultures have been carried out showing very interesting results.

The obtained coating demonstrated effectiveness in tuning the cytotoxicity of chitosan-coated nanocapsules. Moreover, the nanocapsules showed a high degree of internalization in Vero cells, a useful property for the potential application as drug reservoirs directed to cytoplasm release. Finally, the tunability of all the properties of the synthesized chitosan-based nanocapsules was obtained by a very easy and reproducible chemical grafting, indicating that the developed nanocarrier was very promising for further studies oriented toward drug encapsulation for biomedical application.

## 4. Materials and Methods

Tween^®^ 20 (Croda International PLC, Cowick Hall Snaith, Goole, East Yorkshire, UK), absolute ethanol, sodium sulfate anhydrous (99%–100.5%), and sodium chloride (99%), were purchased from Panreac. Span^®^ 85 (sorbitanetrioleate) (Croda International PLC, Cowick Hall Snaith, Goole, East Yorkshire, UK), oleic acid (90%), chitosan (medium molecular weight), Orange II sodium salt, and Bovine Serum Albumine (BSA) were obtained from Sigma-Aldrich. Bis(sulfosuccinimidyl) suberate (BS3) was purchased from Pierce Biotechnology and α-methoxy-ω-amino poly(ethylene glycol) (PEG-MW 5000 Dalton) from IRIS Biotech GmbH. Water (double processed tissue culture) used in all nanocapsule synthesis was from Sigma. Millipore Biomax 300 kDa Ultrafiltration Discs were purchased from Merck Millipore.

Vero cells (monkey kidney epithelial cells) were purchased from the American Type Culture Collection (ATCC, Manassas, VA, USA, number CCL-81). Dulbecco’s modified Eagle’s medium (DMEM), Phosphate-Buffered Saline (PBS), Dulbecco’s Phosphate-Buffered Saline (DPBS) were purchased from Lonza. MTT (3-(4,5-dimethylthiazolyl-2)-2,5-diphenyltetrazolium bromide), penicillin, streptomycin, glutamine solutions, and 4′,6-diamidino-2-fenilindolphenylindol (DAPI) were purchased from Invitrogen (Thermo Fisher Scientific, Waltham, MA, USA).

For the preparation of chitosan-based nanocapsules an organic solution containing 400 mg oleic acid and 86 mg Span^®^ 85 in 40 mL of absolute ethanol was added to the aqueous one, containing 136 mg Tween^®^ 20 solved in 80 mL water, under magnetic stirring during 15 min for the formation of the nanoemulsion. Then 25 mg from a 5 mg/mL chitosan solution in acetic acid 1% (v/v) were added and again the mixture was left under stirring 15 min. Finally, the chitosan-coated nanoemulsion was added to 200 mL of 50 mM Na_2_SO_4_ under sonication (or stirring in the case of optimized nanocapsules). Capsules were separated from Na_2_SO_4_ through ultracentrifugation (30 min, 69673 G, 10 °C), washed with 100 mL of water, centrifuged again, and resuspended in water. The concentration of the nanocapsules in water suspension was obtained by measuring the weight of 1 mL of sample after freeze-drying.

For the grafting of nanocapsule surface suspensions, 20 mg of nanocapsules at a concentration of 2 mg/mL in borate buffer 10 mM pH 8.3 were added with different amounts of the linker bis(sulfosuccinimidyl) suberate (BS3) (20–100 nmol/mgNC) and they were kept under stirring for 30 min. Then a double amount of α-methoxy-ω-amino poly (ethylene glycol) (MeO-PEG-NH_2_) was added and the mixture was kept under stirring for 2 h at 37 °C. Finally, 20 mL of Tris-HCl buffer 10 mM pH 8.0 was added to quench the linker that eventually did not react with PEG. Grafted nanocapsules were filtered using an Amicon Ultrafiltration unit using Millipore Biomax 300 kDa Ultrafiltration Discs to separate them from unreacted PEG. After a washing with fresh water nanocapsules, they were concentrated to a final volume of 2 mL.

Several techniques have been used for the characterization of chitosan nanocapsules.

DLS analysis has been carried out using a Brookhaven 90Plus DLS instrument, by means of the Photo-Correlation Spectroscopy (PCS) technique. Nanoparticle hydrodynamic diameter and polydispersity index (PDI) have been measured in water at the concentration of 0.05 mg/mL.

Electrophoretic mobility (Z Potential) of nanoparticles at different pH values has been determined by measuring the potential of a 0.05 mg/mL nanoparticle suspension in 10 mM KCl with a Plus Particle Size Analyzer (Brookhaven Instruments Corporation).

Nanocapsule composition was analyzed by Fourier Transform Infrared Spectroscopy analysis in a JASCO FT/IR—4100 Fourier transform infrared spectrometer in a frequency range of 600–4000 cm^−1^ with a resolution of 2 cm^−1^ and a scanning number of 32.

Thermogravimetric analysis was performed in a TASTD 2960 thermogravimetric analyzer, by heating the sample at 10 °C/min under air atmosphere.

Environmental Scanning Electron Microscopy (ESEM) images were obtained using a QUANTA-FEG 250 microscope in Scanning Transmission Electron Microscopy (STEM) mode. Bright Field Transmission Electron Microscopy (BF-TEM) analysis was carried out in a FEI Tecnai T20 microscope operating at 200 kV. Due to the sensitive nature of the sample, previous fixation, dehydration, and epoxy resin embedding were necessary for both techniques. This process can be briefly achieved as follows. A fresh sample was synthesized and it was fixed with glutaraldehyde 0.25% in phosphate buffer 10 mM at pH 7.4 for 2 h. It was washed three times with buffer and incubated with 1% osmium tetroxide in PBS for further fixation and staining. Finally, the sample was accurately washed with water and resuspended in 5% gelatin. The sample was centrifuged to obtain a pellet and was incubated overnight at 4 °C. The obtained solid sample was cut in very small pieces before undergoing the subsequent steps. The dehydration of the samples was carried out using the following steps: incubation in ethanol 30%, ethanol 50%, and incubation overnight in ethanol 70%; incubation in ethanol 90% for 1 h; and finally incubation three times in absolute ethanol. After that, samples were incubated overnight in a 1:1 mixture absolute ethanol/epoxy resin (r.t.). The mixture was then removed, changed with absolute epoxy resin and samples were left for impregnation for 8 h at room temperature. After another change of the medium, the final incubation in epoxy resin was carried out overnight at 60 °C to obtain the polymerization.

From different ESEM images (Figures S1 and S2 of Supplementary Materials), an estimation of the diameter distribution has been obtained using Digital Micrograph^®^ (Gatan Inc., Pleasanton, TX, USA) and OriginLab^®^ (OriginLab, Northampton, MA, USA) softwares to measure the diameters of more than 100 nanocapsules and for the frequency count statistical analysis respectively.

Nanocapsule amino content was measured by the Orange II spectrophotometric assay [34,35]. 0.2 mg of nanocapsules were put in contact with 1 mL of 2 mM Orange II sodium salt acidic solution (pH 3) and kept under stirring for 30 min at 37 °C. Capsule suspension was passed through a syringe membrane filter (Millex syringe-driven filter unit, PVDF filter with 0.22 μm pores, purchased from Merck Millipore) to adsorb nanocapsules in the membrane and keep them retained in order to separate them from the Orange II solution. After that, an acidic solution (pH 3) was passed several times through the same filter until all the unbound dye was removed from the nanocapsules (verified by measuring spectrophotometrically the supernatant content). Then they were washed with an alkaline solution (pH 12) to desorb the bound dye from the amino groups on nanocapsules. The washing fractions were collected, the pH was adjusted at 3 and the amount of unadsorbed and desorbed dye was measured at a wave length of 480 nm with a Varian Cary 50 UV/V is spectrophotometer after carrying out a calibration curve.

Resistance of nanocapsules to aggregation has been determined by incubating nanocapsules (3 mL of a 0.15 mg/mL suspension) at different concentrations of albumin from bovine serum (BSA). The hydrodynamic diameter of the capsules under the incubation conditions was measured after 10 min using a Brookhaven 90Plus DLS instrument.

In vitro cell viability test was carried out to determine the cytotoxicity of nanocapsules using 3-(4,5-dimethylthiazol-2)-2,5-diphenyltetrazolium bromide (MTT) colorimetric assay. Vero cells were grown at 37 °C in a 5% CO_2_ atmosphere in Dulbecco’s modified Eagle’s medium (DMEM) supplemented with 10% fetal bovine serum (FBS), penicillin (100 U/mL), streptomycin (100 μg/mL), and glutamine (2 mM). 7500 cells were seeded using a standard 96-well plate (five replicates per sample). After 24 h of incubation in a humidified atmosphere containing 5% CO_2_, the medium was replaced with new medium containing five different concentrations of nanocapsules and a negative control containing no capsules (non-treated cells). After 24 h of incubation, the medium was replaced with fresh medium containing MTT dye solution (0.5 mg/mL in DMEM). After 2 h of incubation at 37 °C and 5% CO_2_, the medium was removed and the formed crystals were dissolved in 200 μL of DMSO. The absorbance was read on a ThermoScientificMultiskan GO TM microplate reader at 570 nm. The relative cell viability (%) related to control cells without nanocapsules was calculated using the percentage ratio between absorbance of the sample and the absorbance of the control. Experiments were carried out in triplicate.

To perform optical microscopy analysis, 3 × 10^4^ cells were seeded on glass coverslips in a 24-well plate at 37 °C. 24 h later, nanocapsules were added at 50 μg/mL in DMEM and incubated for 24 h at 37 °C. Non-internalized nanocapsules were removed, washing with DPBS twice. Cells were fixed with 4% paraformaldehyde for 20 min at 4 °C, washed twice with DPBS, and incubated for 10 min at room temperature with 4′,6-diamidino-2-fenilindolphenylindole (DAPI) for nucleus labeling. The coverslips were mounted on glass microscope slides using ProLong^®^ (Thermo Fisher Scientific Inc., Waltham, MA, USA) Gold Antifade. Optical microscopy was performed using an inverted microscope (Nikon Eclipse Ti-E), and images were analyzed using NIS-Elements Advanced Research software.

## Supplementary Materials

The following are available online at http://www.mdpi.com/1660-3397/14/10/175/s1, Figure S1: ESEM image of nanocapsules, Figure S2: ESEM image of nanocapsules, Figure S3: TGA analysis of nanocapsules, Figure S4: FTIR analysis of grafted nanocapsules, Figure S5: FTIR analysis of starting compounds, Figure S6: FTIR analysis of nanocapsules.

## References

[1] Wilczewska A.Z., Niemirowicz K., Markiewicz K.H., Car H. (2012). Nanoparticles as drug delivery systems. Pharmacol. Rep..

[2] Martinez J.O., Brown B.S., Quattrocchi N., Evangelopoulos M., Ferrari M., Tasciotti E. (2012). Multifunctional to multistage delivery systems: The evolution of nanoparticles for biomedical applications. Chin. Sci. Bull..

[3] Mishra B., Patel B.B., Tiwari S. (2010). Colloidal nanocarriers: A review on formulation technology, types and applications toward targeted drug delivery. Nanomedicine.

[4] Zhang L., Li Y., Jimmy C.Y. (2014). Chemical modification of inorganic nanostructures for targeted and controlled drug delivery in cancer treatment. J. Mater. Chem. B.

[5] Hans M.L., Lowman A.M. (2002). Biodegradable nanoparticles for drug delivery and targeting. Curr. Opin. Solid State Mater. Sci..

[6] Maya S., Sarmento B., Nair A., Rejinold N.S., Nair S.V., Jayakumar R. (2013). Smart stimuli sensitive nanogels in cancer drug delivery and imaging: A review. Curr. Pharm. Des..

[7] Kawaguchi H. (2014). Thermoresponsive microhydrogels: Preparation, properties and applications. Polym. Int..

[8] Karimi M., Sahandi Zangabad P., Ghasemi A., Amiri M., Bahrami M., Malekzad H., Ghahramanzadeh Asl H., Mahdieh Z., Bozorgomid M., Ghasemi A. (2016). Hambling, Temperature-responsive smart nanocarriers for delivery of therapeutic agents: Applications and recent advances. ACS Appl. Mater. Interfaces.

[9] Allen T.M., Cullis P.R. (2013). Liposomal drug delivery systems: From concept to clinical applications. Adv. Drug Deliv. Rev..

[10] Natarajan J.V., Nugraha C., Ng X.W., Venkatraman S. (2014). Sustained-release from nanocarriers: A review. J. Control. Release.

[11] Shimanovich U., Bernardes G.J.L., Knowles T.P.J., Cavaco-Paulo A. (2014). Protein micro-and nano-capsules for biomedical applications. Chem. Soc. Rev..

[12] Vrignaud S., Benoit J.P., Saulnier P. (2011). Strategies for the nanoencapsulation of hydrophilic molecules in polymer-based nanoparticles. Biomaterials.

[13] Solans C., Izquierdo P., Nolla J., Azemar N., Garcia-Celma M.J. (2005). Nano-emulsions. Curr. Opin. Colloid Interface Sci..

[14] Bouchemal K., Briançon S., Perrier E., Fessi H. (2004). Nano-emulsion formulation using spontaneous emulsification: Solvent, oil and surfactant optimisation. Int. J. Pharm..

[15] Anton N., Benoit J.P., Saulnier P. (2008). Design and production of nanoparticles formulated from nano-emulsion templates—A review. J. Control. Release.

[16] Soppimath K.S., Aminabhavi T.M., Kulkarni A.R., Rudzinski W.E. (2001). Biodegradable polymeric nanoparticles as drug delivery devices. J. Control. Release.

[17] Kumari A., Yadav S.K., Yadav S.C. (2010). Biodegradable polymeric nanoparticles based drug delivery systems. Colloids Surf. B Biointerfaces.

[18] Zimmer A., Kreuter J. (1995). Microspheres and nanoparticles used in ocular delivery systems. Adv. Drug Deliv. Rev..

[19] Sundar S., Kundu J., Kundu S.C. (2010). Biopolymeric nanoparticles. Sci. Technol. Adv. Mater..

[20] Huang S., Fu X. (2010). Naturally derived materials-based cell and drug delivery systems in skin regeneration. J. Control. Release.

[21] Younes I., Rinaudo M. (2015). Chitin and chitosan preparation from marine sources. Structure, properties and applications. Mar. Drugs.

[22] Prabaharan M. (2015). Chitosan-based nanoparticles for tumor-targeted drug delivery. Int. J. Biol. Macromol..

[23] Nagpal K., Singh S.K., Mishra D.N. (2010). Chitosan nanoparticles: A promising system in novel drug delivery. Chem. Pharm. Bull..

[24] Chen C.K., Wang Q., Jones C.H., Yu Y., Zhang H., Law W.C., Lai C.K., Zeng Q., Prasad P.N., Pfeifer B.A. (2014). Synthesis of pH-responsive chitosan nanocapsules for the controlled delivery of doxorubicin. Langmuir.

[25] Felt O., Buri P., Gurny R. (1998). Chitosan: A unique polysaccharide for drug delivery. Drug Dev. Ind. Pharm..

[26] Onoue S., Yamada S., Chan H.K. (2014). Nanodrugs: Pharmacokinetics and safety. Int. J. Nanomed..

[27] Lehner R., Wang X., Marsch S., Hunziker P. (2013). Intelligent nanomaterials for medicine: Carrier platforms and targeting strategies in the context of clinical application. Nanomedicine.

[28] Nicolas J., Mura S., Brambilla D., Mackiewicz N., Couvreur P. (2013). Design, functionalization strategies and biomedical applications of targeted biodegradable/biocompatible polymer-based nanocarriers for drug delivery. Chem. Soc. Rev..

[29] Kyzas G.Z., Bikiaris D.N. (2015). Recent modifications of chitosan for adsorption applications: A critical and systematic review. Mar. Drugs.

[30] Moghimi S.M., Hunter A.C., Murray J.C. (2001). Long-circulating and target-specific nanoparticles: Theory to practice. Pharmacol. Rev..

[31] Zhang Y., Chan H.F., Leong K.W. (2013). Advanced materials and processing for drug delivery: The past and the future. Adv. Drug Deliv. Rev..

[32] Sapsford K.E., Algar W.R., Berti L., Gemmill K.B., Casey B.J., Oh E., Stewart M.H., Medintz I.L. (2013). Functionalizing nanoparticles with biological molecules: Developing chemistries that facilitate nanotechnology. Chem. Rev..

[33] Rabanel J.M., Hildgen P., Banquy X. (2014). Assessment of PEG on polymeric particles surface, a key step in drug carrier translation. J. Control. Release.

[34] Klibanov A.L., Maruyama K., Torchilin V.P., Huang L. (1990). Amphipathic polyethyleneglycols effectively prolong the circulation time of liposomes. FEBS Lett..

[35] Maruyama K., Yuda T., Okamoto A., Ishikura C., Kojima S., Iwatsuru M. (1991). Effect of molecular weight in amphipathic polyethyleneglycol on prolonging the circulation time of large unilamellar liposomes. Chem. Pharm. Bull..

[36] Tavares I.S., Caroni A.L.P.F., Dantas-Neto A.A., Pereira M.R., Fonseca J.L.C. (2012). Surface charging and dimensions of chitosan coacervated nanoparticles. Colloids Surf. B Biointerfaces.

[37] Berthold A., Cremer K., Kreuter J. (1996). Preparation and characterization of chitosan microspheres as drug carrier for prednisolone sodium phosphate as model for antiinflammatory drugs. J. Control. Release.

[38] Mao H.-Q., Roy K., Troung-Le V.L., Janes K.A., Lin K.Y., Wang Y., August J.T., Leong K.W. (2001). Chitosan-DNA nanoparticles as gene carriers: Synthesis, characterization and transfection efficiency. J. Control. Release.

[39] Arenal R., De Matteis L., Custardoy L., Mayoral A., Tence M., Grazú V., De La Fuente J.M., Marguina C., Ibarra M.R. (2013). Spatially-resolved EELS analysis of antibody distribution on bio-functionalized magnetic nanoparticles. ACS Nano.

[40] Noel S., Liberelle B., Robitaille L., De Crescenzo G. (2011). Quantification of primary amine groups available for subsequent biofunctionalization of polymer surfaces. Bioconjug. Chem..

[41] Thierry B., Griesser H.J. (2012). Dense PEG layers for efficient immunotargeting of nanoparticles to cancer cells. J. Mater. Chem..

[42] Moros M., Mitchell S.G., Grazú V., Tence M., De La Fuente J.M. (2013). The fate of nanocarriers as nanomedicines in vivo: Important considerations and biological barriers to overcome. Curr. Med. Chem..

[43] Duncan R. (2011). Polymer therapeutics as nanomedicines: New perspectives. Curr. Opin. Biotechnol..

