# Supplementary material for: Controlling Properties and Cytotoxicity of Chitosan Nanocapsules by Chemical Grafting
Source: Mar Drugs. 2016 Sep 30;14(10):175. doi: 10.3390/md14100175 (PMC5082323; doi:10.3390/md14100175)
Supplement: Supplementary file 1 [file marinedrugs-14-00175-s001.pdf]

Supplementary Materials: Controlling Properties and Cytotoxicity of Chitosan Nanocapsules by Chemical Grafting

Laura De Matteis, Maria Alleva, Inés Serrano-Sevilla, Sonia García-Embid, Grazyna Stepien, María Moros, and Jesús M. de la Fuente

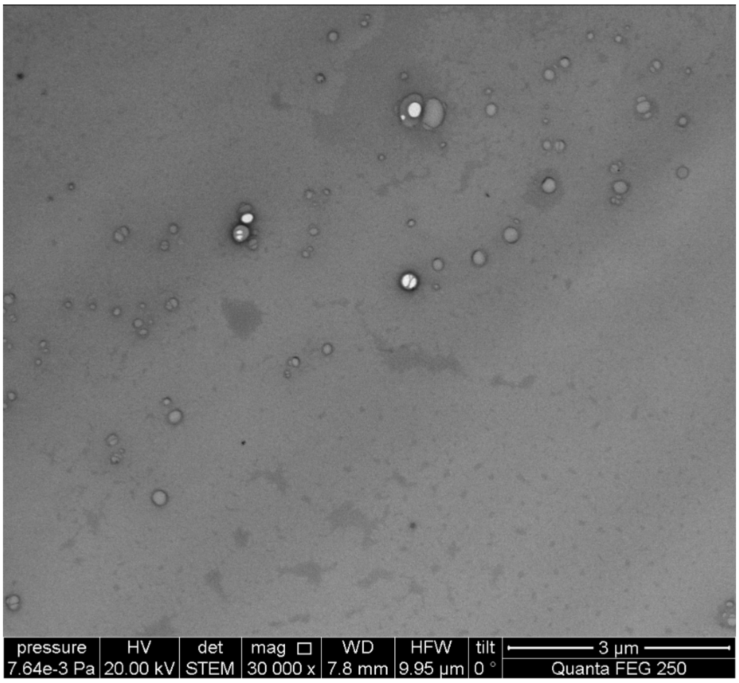

Figure S1. ESEM images of nanocapsules from a section of the epoxy resin block.

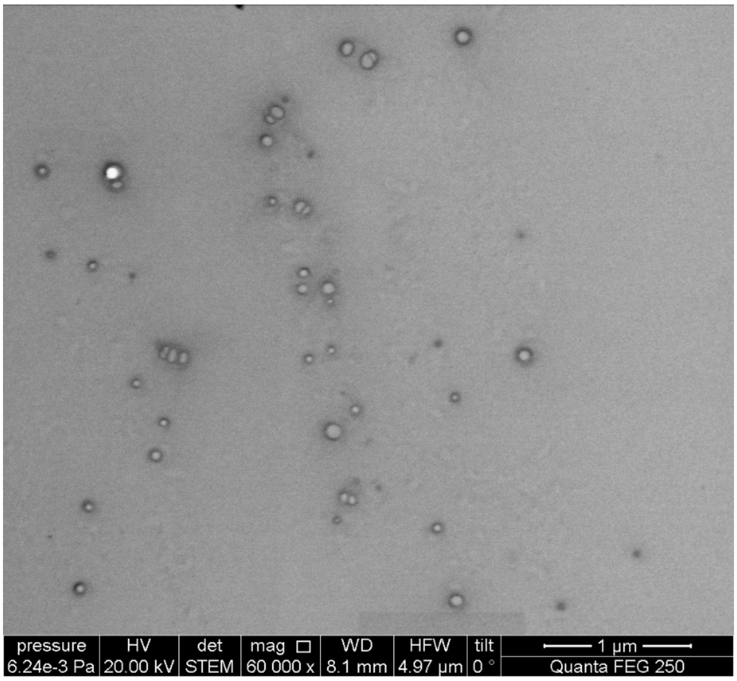

Figure S2. ESEM images of nanocapsules from a section of the epoxy resin block.

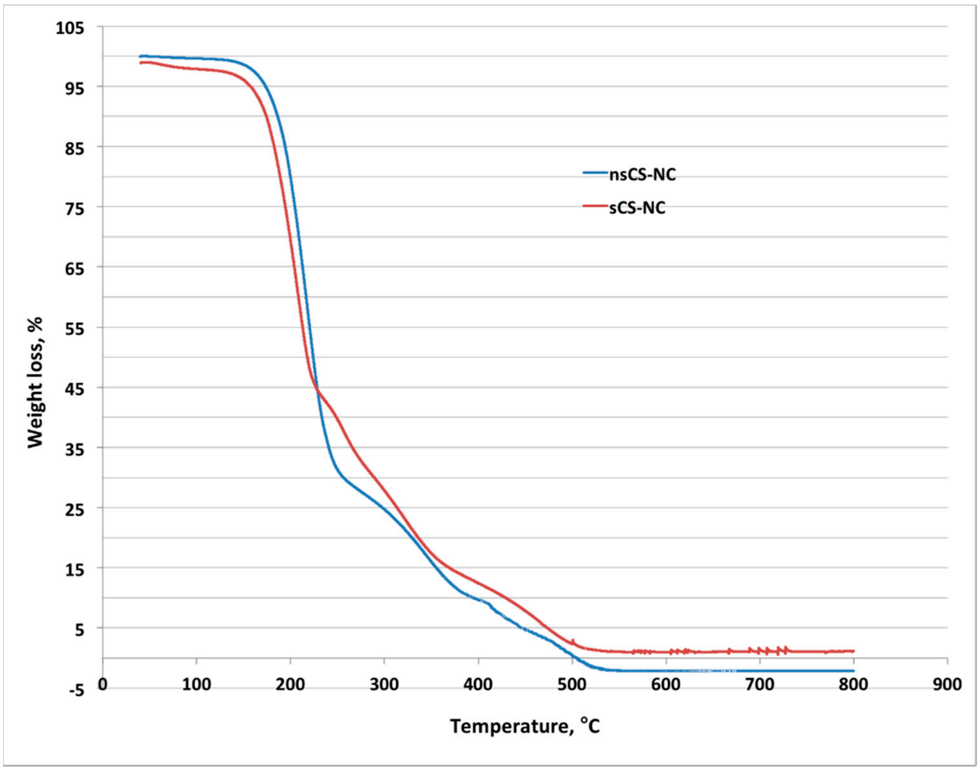

Figure S3. TGA analysis of sonicated (sCS-NC) and not sonicated (ns-CS-NC) chitosan nanocapsules.

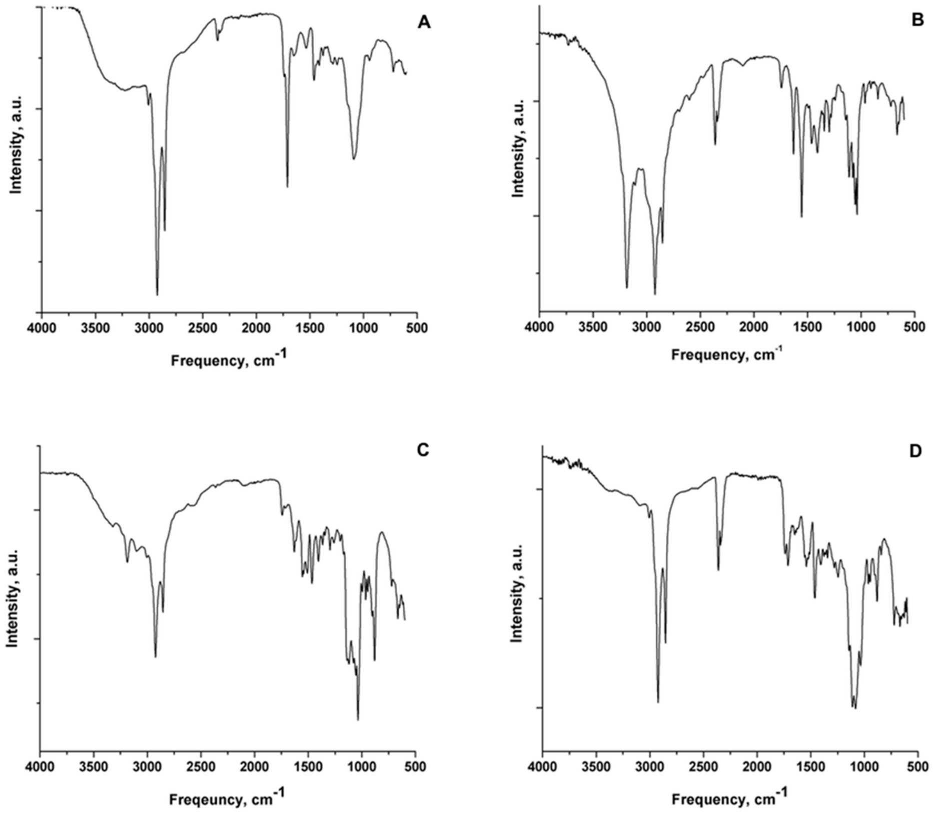

Figure S4. FTIR analysis of nsCS-NC before grafting (**A**); after BS^3 linking (intermediate step) (**B**) and after grafting with different amounts of MeO-PEG5000-NH_2: ldCS-NC (**C**) and hdCS-NC (**D**).

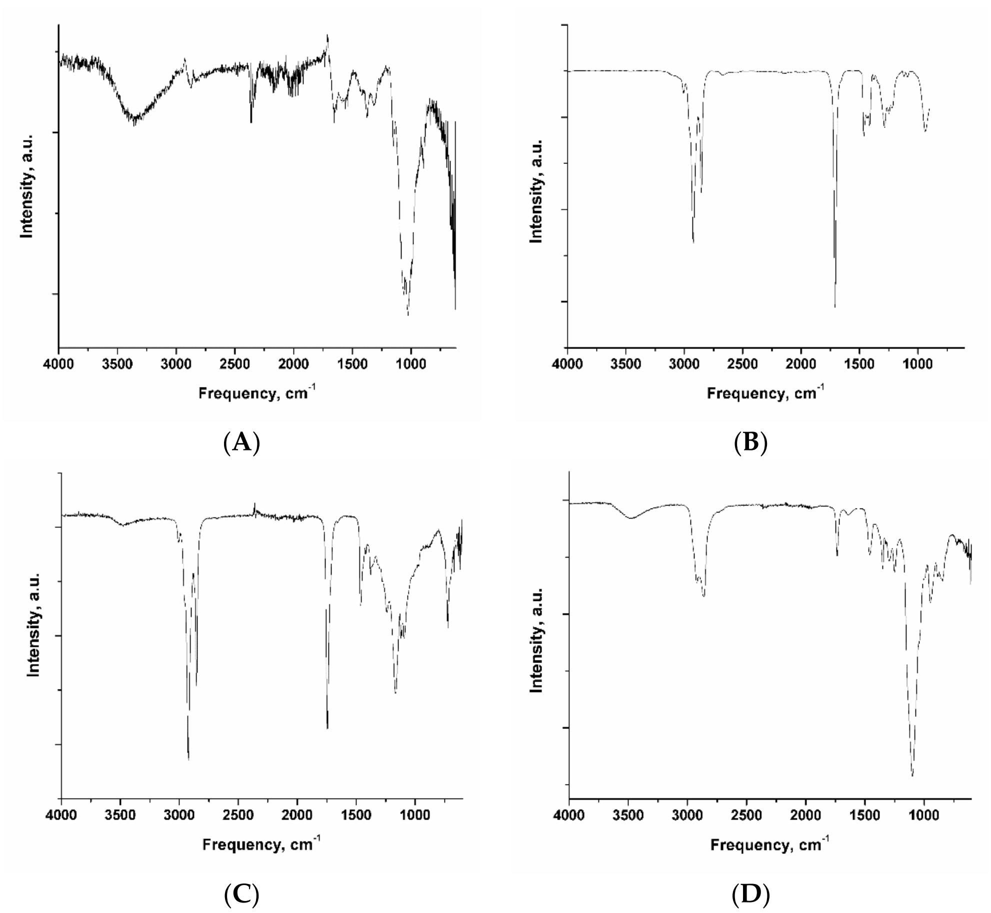

Figure S5. FTIR analysis of chitosan (**A**), oleic acid (**B**), Span 85 (**C**) and Tween 20 (**D**).

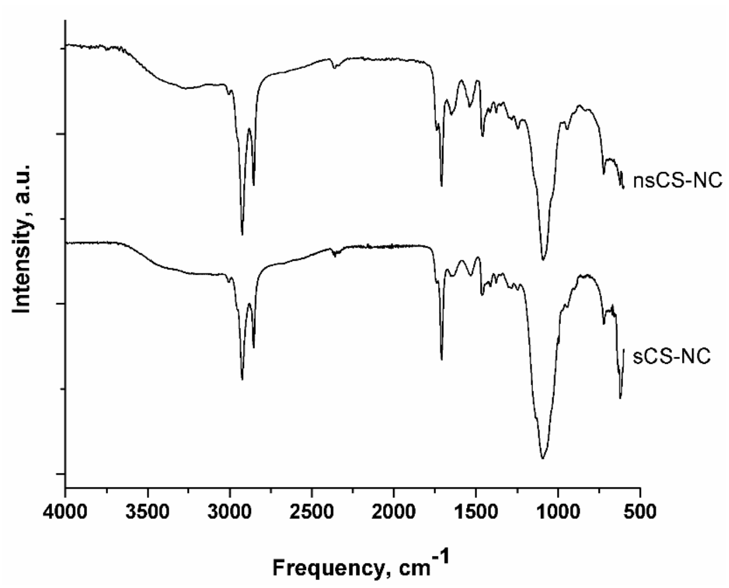

Figure S6. FTIR analysis of sonicated (sCS-NC) and not sonicated (ns-CS-NC) chitosan nanocapsules.
